# Early infant appetitive traits are associated with growth status and adiposity in African-American infants and toddlers

**DOI:** 10.1080/03014460.2025.2557266

**Published:** 2025-10-02

**Authors:** Babette S. Zemel, Gary D. Wu, Eileen Ford, Ceylan Tanes, Patricia A. DeRusso, Andrea Kelly

**Affiliations:** a Division of Gastroenterology, Hepatology and Nutrition, The Children’s Hospital of Philadelphia, Philadelphia, Pennsylvania, USA; b Department of Pediatrics, Perelman School of Medicine, University of Pennsylvania, Philadelphia, Pennsylvania, USA; c Division of Gastroenterology, Perelman School of Medicine, University of Pennsylvania, Philadelphia, Pennsylvania, USA; d Division of Endocrinology and Diabetes, Children’s Hospital of Philadelphia, Philadelphia, Pennsylvania, USA

**Keywords:** Eating behaviour, infant, maternal obesity, appetite, adiposity

## Abstract

**Background:**

Few studies have examined infant appetitive traits in minority populations or among mothers with obesity.

**Aim:**

To test associations of appetitive traits with nutritional exposures and growth in African-American children up to age 24m.

**Subjects and methods:**

Mothers with pre-pregnancy BMI <25 or ≥30 and their infants were included (*n* = 222). The Baby Eating Behaviour Questionnaire (BEBQ) at 3m captured appetitive traits; BMI z-scores (BMIZ) at 3, 12 and 24m were examined. Internal consistency of BEBQ constructs was tested and their association with growth outcomes evaluated using correlation analysis, multiple regression, and tests for trend.

**Results:**

Only the BEBQ construct “food responsiveness” met internal consistency criteria (Cronbach’s alpha = 0.85). “General appetite” and single items serving as proxies for “slowness in eating,” “enjoyment of food” and “satiety responsiveness” associated with growth outcomes. Food responsiveness was higher for mixed vs. formula-fed infants (*p* = 0.004), infants breastfed >3m (*p* = 0.006), and infants whose mothers reported food insecurity (*p* = 0.04). Multiple individual appetitive trait items associated with BMIZ at 24m, even after adjustment for baseline BMIZ (3m), maternal obesity status and addition of cereal to bottles.

**Conclusions:**

Infant appetitive traits prospectively associate with relative weight and adiposity at 24m and aspects of the postnatal nutritional environment independent of maternal BMI.

## Introduction

The first two years of life are critical for shaping long-term health (Whitaker and Dietz 1998; Mameli et al. 2016; Schwarzenberg and Georgieff 2018). Disparities in risk factors (Taveras et al. 2013) operating in this period (Young et al. 2012; Woo Baidal et al. 2016; Ahluwalia 2020) may contribute to the disproportionate prevalence of obesity among Black and Hispanic youth in the US (Hales et al. 2017). For example, excess or rapid infant weight gain in the first two years of life associate with subsequent obesity risk and group disparities (Stettler et al. 2003; Baird et al. 2005; Monteiro and Victora 2005; Taveras et al. 2009; Woo Baidal et al. 2016; Taveras et al. 2010; Isong et al. 2018; Zheng et al. 2018). African-American infants are less likely to breastfeed than Hispanic or white infants, and more likely to receive solid foods at <4m of age (Taveras et al. 2010; Roess et al. 2018; Thompson and Bentley 2013). Other dimensions of the broader food environment include food insecurity, which is more common in non-Hispanic Black and Hispanic households than in non-Hispanic White households (Odoms-Young and Bruce 2018). Food insecurity can impact a myriad of food related behaviours and choices, such as dietary diversity, controlling feeding practices and maladaptive feeding behaviours (Arlinghaus and Laska 2021). Maternal obesity, which is also more prevalent among African-American women, is a strong predictor of obesity development in offspring, and potential pathways of influence include the intrauterine environment, postnatal nutritional environment, and shared genetics. Few studies have examined infant appetitive behaviours in the context of maternal obesity. Appetitive traits expressed early in infancy are associated with current and subsequent weight, but the role of early life appetite traits on weight status is relatively unexplored in African-American infants and children.

The Baby Eating Behaviour Questionnaire (BEBQ) is a tool for assessing parent-perceived appetite traits and eating behaviours of milk-feeding infants. It is designed to characterise five appetite traits: “enjoyment of food” (perceived liking of milk and feeding), “food responsiveness” (level of demand for feeds and degree of responsiveness to milk and feeding cues), “slowness in eating” (typical speed of feeding), “satiety responsiveness” (ease of reaching fullness during a feed), and “general appetite” (degree to which the infant is considered to have a big appetite) (Llewellyn et al. 2011; Kininmonth et al. 2021; Oyama et al. 2021; Schneider-Worthington et al. 2021; Vandyousefi et al. 2021; Barrett and Thompson 2022; Sanjeevi et al. 2022; Vandyousefi et al. 2022). Infant appetitive traits have been associated with variation in body size and adiposity in the first two years of life (Wright et al. 2006; van Jaarsveld et al. 2011, 2014; Quah et al. 2015; Kininmonth et al. 2021; Barrett and Thompson 2022; Vandyousefi et al. 2021; Agras et al. 1987).

The BEBQ was developed in the United Kingdom in the Gemini twin birth cohort study (Llewellyn et al. 2011; van Jaarsveld et al. 2010), and has been used in a number of studies in other populations (Oyama et al. 2021; Vandyousefi et al. 2022; Quah et al. 2015; Mallan et al. 2014). Only one published analysis of BEBQ data included a predominantly African-American cohort, and the sample was small and cross-sectional (Shepard and Chandler-Laney 2015). It is unknown whether infant appetite traits associate with early risk factors for obesity that disproportionately impact African-Americans, including lower rate and shorter duration of breastfeeding, early introduction to solid foods, food insecurity and maternal obesity (Taveras et al. 2010; Roess et al. 2018; Thompson and Bentley 2013). In addition, the internal consistency of the BEBQ constructs have not previously been assessed in an African-American cohort. Examining internal consistency was considered an important preliminary step in the current analysis given that the BEBQ was developed in Britain in a largely white cohort (Llewellyn et al. 2011; van Jaarsveld et al. 2010).

This study examined BEBQ responses among African-American mother-infant dyads recruited in an urban health care setting in the U.S. After assessing the factor structure of the BEBQ in this sample, associations were tested between infant appetitive traits, intrauterine and other nutritional environment exposures (*i.e.* pregnancy weight gain, infant birth weight, milk feeding practice (breastmilk, formula or mixed), timing of introduction to solids, frequency of adding cereal to the bottle, food insecurity), and growth and adiposity markers (weight- and body mass index (BMI)-for-age z-scores, skinfold thicknesses) in the first 2 years of life. Mothers were recruited into a group with healthy weight or a group with obesity based on early pregnancy BMI (<25 kg/m^2^ or ≥30 kg/m^2^, respectively), and differences in appetitive trait scores according to maternal weight status were assessed.

We hypothesised that food approach traits (*i.e.* enjoyment of food, food responsiveness, general appetite) would associate positively with BMI-for-age and weight-for-age z-scores and skinfold thicknesses, while food avoidant traits (*i.e.* slowness in eating, satiety responsiveness) would associate negatively with these same outcomes. Further, we hypothesised that these associations would be sustained longitudinally to 24m of age and be independently associated with growth and body composition outcomes after adjusting for covariates. Given the paucity of information on associations of maternal weight status and other intrauterine and postnatal nutritional environment exposures with infant appetitive traits, we also conducted exploratory analyses to more fully describe contextual factors that may associate with appetitive traits in this cohort.

## Materials and methods

### Study design and participants

This was a secondary analysis of data from the prospective, longitudinal *Infant Growth and Microbiome Study* (IGram) conducted at Children’s Hospital of Philadelphia (CHOP). Enrolment began in 2014 and the last 24-month visit was completed in 2019. Pregnant women self-identifying as African-American were enrolled in their 3^rd^ trimester if their early-pregnancy BMI (recorded at ≤18 weeks gestation and extracted from medical records) was <25 kg/m^2^ (group with healthy weight) or ≥30 kg/m^2^ (group with obesity). Mothers with a singleton pregnancy who did not have medical conditions associated with obesity or glucose regulation, uncontrolled thyroid disease, or chronic inflammatory or autoimmune disease, and who did not take certain medications (Bittinger et al. 2020), were eligible for inclusion.

Eligible infants were those born to enrolled mothers at the Hospital of the University of Pennsylvania at ≥37 weeks gestation, with an appropriate size for their gestational age, and without major congenital malformations, foetal abnormalities, or significant illness that might have impacted their growth and development. The target sample size was 300 mother-infant dyads likely to remain in the study for 24m (accounting for attrition). CHOP’s Committee for the Protection of Human Subjects (Internal Review Board) reviewed and approved the study protocol (IRB 14–010833) and informed consent was given by all enrolled mothers.

### Data collection

Data on demographic and socioeconomic factors (age, race, education, income), medical history, and household food insecurity were collected by questionnaire at the 3^rd^ trimester visit. The first two questions of the United States Department of Agriculture food insecurity questionnaire were used to assess food insecurity (“we worried whether our food would run out before we got money to buy more” and “the food that we bought just didn’t last, and we didn’t have money to get more”; Hager et al. 2010). Possible answers were “never true,” “sometimes true,” and “often true.” Food insecurity was defined if either of the latter two responses were selected.

Gestational weight gain was obtained from medical records as the difference between earliest maternal weight measured before 18 weeks gestation, and last measured weight prior to delivery. Information on gestational age at birth and birth complications was collected from the medical record, and weight and length were measured at the birth visit (within 1 to 4 days of delivery). Study visits occurred at birth and months 1, 2, 3, 4, 6, 9, 12, 15, 18, 21, and 24. The current study used data collected in the 3^rd^ trimester, at birth, and at months 3, 12, and 24.

Infant anthropometric measurements were obtained in triplicate by trained study personnel following the standard techniques (Lohman et al. 1988) and the average was used in analyses. Recumbent length was measured to 0.1cm using an infant length board (Harpenden, Crymych, UK), weight to 0.01kg without clothing or diaper using a digital electronic scale (Scaletronix, White Plains, NY), and biceps, triceps, subscapular, and suprailiac skinfold thicknesses to 0.1mm using skinfold callipers (Holtain, Crymych, UK). Infant weight and length values were used to calculate weight-for-age (WAZ), length-for-age (HAZ) and BMI-for-age z-scores (BMIZ) based on the World Health Organization Multicentre Growth Reference study (WHO Multicentre Growth Reference Study Group, 2012). Biceps, triceps, subscapular, and suprailiac skinfold thicknesses were summed, and the sum of skinfolds variable was used in analyses (hereafter referred to as “skinfolds”).

Milk feeding practices (*i.e.* exclusive breastfeeding, exclusive formula feeding, or mixed breast and formula feeding) and introduction of complementary foods were assessed at all visits using items from the Centers for Disease Control and Prevention’s (CDC) Infant Feeding Practices Study II questionnaire (Fein et al. 2008). The data collection period pre-dated the World Health Organization standardised definitions of infant feeding practices, so our terms do not precisely conform to those definitions (World Health Organization 2021). With respect to milk feeding, mothers were asked: “how are you currently feeding your baby milk?” Possible answers were “breastfeeding only,” “both breast and formula feeding,” “formula feeding only,” “other milk,” and “no milk.” Responses at the 3m visit were used in the analysis as “milk feeding practices at 3m.” Additional aspects of milk feeding were captured for analyses with an exclusive breastfeeding variable (operationalised as never, between 1 and 6m, or >6m); and a continuous variable to capture the duration of any breastfeeding (either exclusively or mixed) in months. Because the latter variable was heavily skewed with many zero values, it was categorised as mothers who breastfed their infant up to 3m of age (when the BEBQ was administered) vs. mothers who breastfed >3m of age.

Each month, mothers were asked, “since your last visit, have you started your baby on solid foods for the very first time?” A “first food month” variable took on integer values reflecting the first month mothers reported introducing one or more complementary foods to their child. At the 3m visit, mothers/caregivers were asked: “how often have you added baby cereal to your baby’s bottle of formula, pumped/expressed breast milk, or other milk in the past 2 weeks?” Possible answers were “never,” “only rarely,” “every few days,” “about once a day,” “at most feedings,” and “every feeding.” Frequency of cereal added to the bottle at 3m was operationalised as a binary variable, with responses of “every few days,” “about once a day,” “at most feedings,” and “every feeding” coded as frequent.

### Infant appetitive traits

The BEBQ was administered by interview at the 3m visit. It is an 18-item instrument designed to assess infant appetite and eating behaviour traits during the period of exclusive breast and/or formula feeding (Llewellyn et al. 2011). The questionnaire was designed to capture five appetitive trait constructs: (1) *enjoyment of food* based on four items (e.g. “my baby loves milk”); (2) *food responsiveness* based on six items (e.g. “my baby is always demanding a feed”); (3) *slowness in eating* based on four items (e.g. “my baby feeds slowly”); (4) *satiety responsiveness* based on three items (e.g. “my baby finds it difficult to manage a complete feed”); and (5) *general appetite* based on the single item “my baby has a big appetite.” This questionnaire correlated with all of the constructs in Llewellyn et al.’s (Llewellyn et al. 2011) original study and is meant to capture general appetite. Response options for each of the 18 items were on a Likert-type scale ranging from “never” (1) to “always” (5) following the prompt, “how would you describe your baby’s feeding style at a typical daytime feeding?” To derive a score for each appetitive trait construct, item responses were summed and then divided by the number of items in that construct. In each case, a higher score denotes greater expression of that appetitive trait, as perceived by the parent completing the questionnaire.

### Statistical analysis

As this is a secondary analysis of IGram study data, a power analysis specific to these analyses was not conducted. BMIZ at 24m of age was the primary outcome of interest because it is the best anthropometric indicator of weight relative to overall body size (Roy et al. 2016), and because the goal was to examine the long-term association of early infant appetitive traits with growth outcomes. Therefore, the analytic sample was comprised of cases with complete BEBQ data at 3 months and BMIZ at 24 months. HAZ, WAZ and skinfolds were secondary outcomes.

Confirmatory factor analysis with the lavaan package (Rosseel 2012) (v.0.6–11) in R was used to test whether the original BEBQ four-factor model fit well to the data for this cohort. Following Mallan et al. (Mallan et al. 2014) and Hu and Bentler (Hu and Bentler 1999), model fit was assessed using normed chi-square (i.e. the chi-square statistic divided by degrees of freedom), Tucker-Lewis (TLI) and Comparative Fit (CFI) indices, and the root mean-square error of approximation (RMSEA). A normed chi-square value between 1 and 2, values at or above 0.90 for TLI and CFI, and RMSEA ≤0.06 indicate good model fit. The Cronbach’s Alpha statistic was used to assess internal consistency of BEBQ constructs, with values (between 0 and 1) of 0.70 or above generally considered acceptable (Tavakol and Dennick 2011).

To assess for potential bias in the sample, we compared the characteristics of the infants in the analytic sample with those infants enrolled who did not meet the criteria for inclusion in this analysis, i.e. did not have complete data for the BEBQ questionnaire at 3m or BMIZ at 24m. We compared characteristics collected at birth of these two groups since some of the infants not included in the analytic sample were lost to follow-up before the 3m visit and thus do not have data for comparison. Within the analytic sample, we also compared the characteristics of mother-infant dyads with 1 or more missing data points for any variable.

Maternal age, maternal weight status, early pregnancy weight, gestational weight gain, birth weight, gestational age, exclusive breastfeeding, milk feeding type at 3m, breastfeeding beyond 3m, age at introduction to solid foods, and frequency of cereal added to the bottle at 3m were considered as potential confounders in models predicting BMIZ, WAZ, or skinfolds. None of these variables were significantly associated with both the independent (appetite) and dependent (growth) outcomes of interest. Because of the known effects of milk feeding practices and maternal weight status on child weight outcomes in the general population (Heslehurst et al. 2019; Horta et al. 2015), these variables were included in multivariable models predicting primary and secondary outcomes.

Statistical analyses were performed using R (R Core Team, 2022) (v.4.2.1) in RStudio (v.2022.02.3) or Stata (v18.0, College Station, TX). All tests were two-sided, and results were considered significant at *p* < 0.05. Data were described using mean or median for continuous variables and counts and percentages for categorical variables. To examine the degree of association between continuous variables such as food responsiveness vs BMIZ, Spearman correlations were used. To test for differences in continuous outcomes between independent groups, independent sample t-tests were used. To test for differences in proportion between two categorical variables, such as BEBQ general appetite vs maternal weight status, chi-squared test was used. To test the relationship between continuous (e.g. BMIZ) and categorical variables (e.g. feeding mode), ANOVA with Bonferroni correction was used.

To formally test for trends in BMIZ, WAZ, and skinfold variables across ordinal appetite trait variables, Cuzick’s test, an extension of the Wilcoxon rank-sum test (Cuzick 1985) was used. In multivariable models, marginal means and their 95% confidence intervals (emmeans (Lenth et al. 2022) v.1.8.2) were estimated for BMIZ, WAZ, and skinfolds across levels of ordinal appetite variables, adjusting for BMIZ, WAZ, or skinfolds at 3m (to account for the infant’s growth trajectory), maternal weight status, and milk feeding practices at 3m. This included a formal test for trend in the marginal means. Finally, we used stepwise regression analysis to determine whether appetitive traits or combinations of relevant appetitive traits associated with BMIZ, WAZ, and skinfolds after accounting for values at age 3m. The stepwise model used a probability of retention of *p* ≤ 0.05 and considered the following variables: maternal obesity, pregnancy weight gain, birthweight Z-scores, feeding mode at 3m (breastmilk, formula or both), duration of breastfeeding for 3 or more months, timing of introduction of solids, how frequently cereal was added to the bottle at 3m, and food insecurity.

## Results

### Participant characteristics

**Figure 1.**
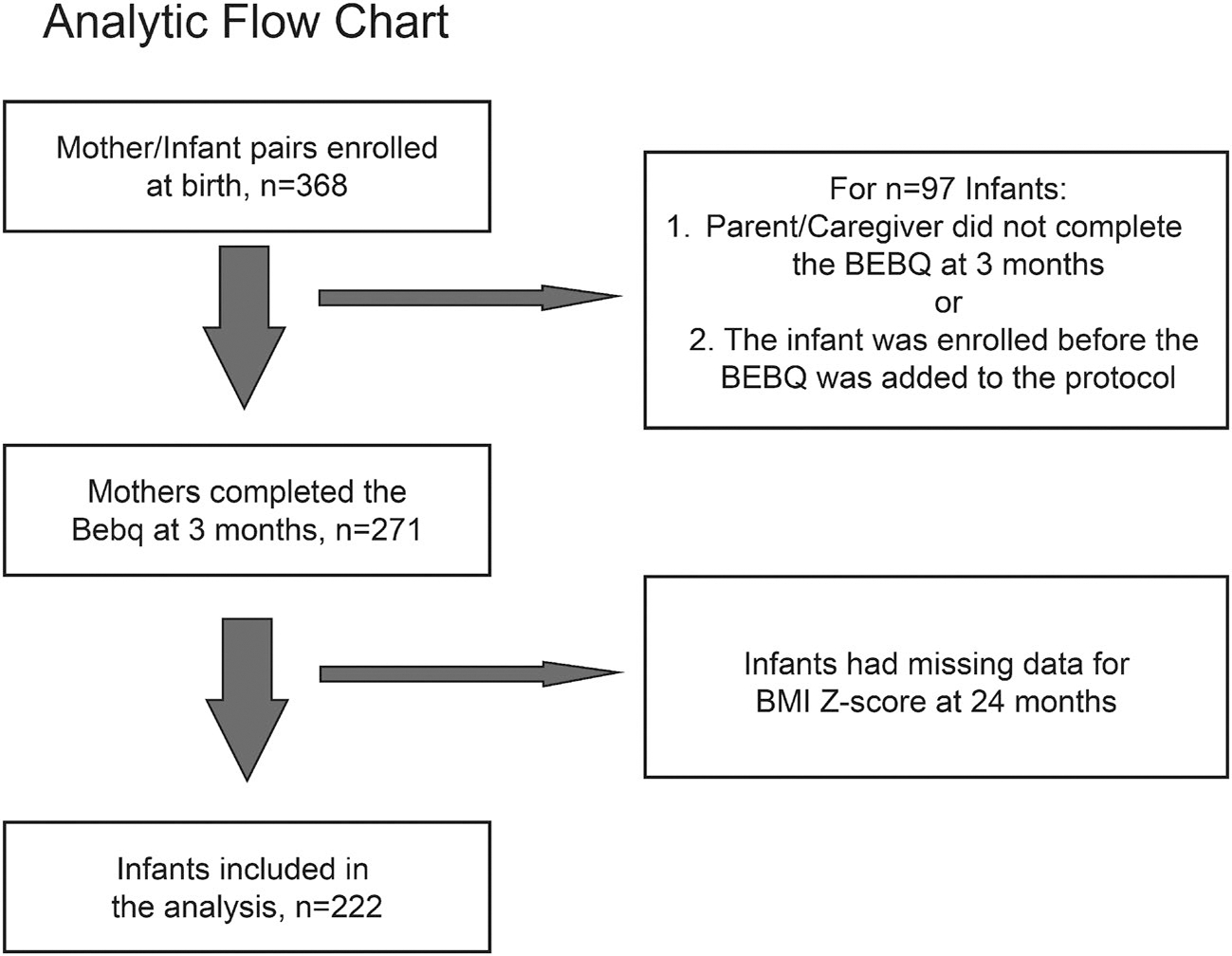
Infant growth and Microbiome Study (IGram) analytic sample flowchart.

The BEBQ questionnaire was added to the protocol after the IGram study’s initiation. Of the 368 infants enrolled at birth, 222 infants had complete data for the BEBQ at 3m and BMIZ at 24m (Figure 1). The BEBQ was completed by the mother for 219 infants and by the father for 3 infants. Forty-nine completed the BEBQ at 3m but did not have BMIZ data at 24m. Baseline characteristics of the analytic sample did not differ from the subgroup with complete data (*n* = 146) (Supplemental Table 1), nor did the characteristics of mother-infant dyads who had at least one missing data element (Supplemental Table 2) except for sum of skinfolds at 12 months.

Characteristics of the analytic sample are given in Table 1. Forty-six percent of mothers were in the healthy weight group. Most mothers had an annual household gross income <$50,000, and 37% reported experiencing some degree of food insecurity in their 3^rd^ trimester. At the 3m visit, 20% of infants were exclusively breastfed, 57% were receiving formula, and 23% were receiving both. Forty one percent of mothers continued to breastfeed beyond 3m, either exclusively or with supplementary formula. Mean ± SD age of introduction to complementary foods was 3.6 ± 1.7m, and at 3m, 30% of mothers were frequently adding cereal to their infant’s bottle.

### BEBQ confirmatory factor analysis

The original BEBQ four-factor structure (Llewellyn et al. 2011) (excluding the single factor “*general appetite”)* fit poorly to the data (X^2^/df = 1.73); CFI = 0.91; TLI = 0.89; RMSEA = 0.06). Cronbach’s α was close to acceptable for the food responsiveness appetitive trait construct (α = 0.84, 6 items), but ranged from unacceptable to poor for the others (*enjoyment of food* α = 0.39, 4 items; *satiety responsiveness* α = 0.46, 3 items; *slowness in eating* α = 0.51, 3 items). The single item appetitive trait, *general appetite*, had 4 responses of “never,” so the responses “never” and “rarely” were combined for use in analyses.

### Associations between BEBQ measures and growth/adiposity markers

Although not all BEBQ trait constructs reached an acceptable level of internal consistency, we conducted exploratory analysis to evaluate the relationship of each appetitive trait construct and individual BEBQ questionnaire items with growth outcomes using Spearman correlations (Table 2). HAZ at 24m was not associated with BEBQ trait constructs or individual items, so we did not continue to analyse this measure of growth. Based on the significant correlations with BMIZ, WAZ and sum of skinfolds at 24m (Table 2), we carried forward individual BEBQ items for further analysis. The distributions of the appetitive trait constructs and selected individual item scores are shown in Table 3.

*Food responsiveness*, the only multi-item appetitive trait construct that demonstrated internal consistency, was not associated with growth or adiposity outcomes at any age. *General appetite,* a single item construct, was positively correlated with BMIZ and sum of skinfolds at 12 and 24m of age (rho range from 0.15 to 0.21). The 4-item appetitive trait construct, *Enjoyment of food*, was inversely associated with BMIZ at 12 and 24m, and WAZ at 24m (rho range from −0.14 to −0.15); this correlation was largely driven by 2 items (“My baby becomes distressed while feeding” which is inversely scored, and “My baby enjoys feeding time”) as shown in Table 2. Because the baby distress item was the one correlated with BMIZ at 24m we carried it forward for additional analyses. Of note, only 3 mothers reported that their baby always/often becomes distressed when feeding, so these categories were combined with sometimes becomes distressed while feeding.

The 3-item appetitive trait construct, *satiety responsiveness*, was also significantly and inversely associated with sum of skinfolds at 3 and 24m (rho range from −0.14 to −0.16, Table 2); this association was largely driven by the item, “My baby gets full before taking all the milk I think he/she should have.” This single item was carried forward for additional analyses, and the categories “often” and “always” were combined for the purposes of analysis.

Interestingly, the 4-item appetitive trait construct, *slowness in eating*, showed consistent inverse correlations with growth and adiposity outcomes at multiple ages (rho range from −0.15 to −0.23), and the single question, “My baby feeds slowly” was significantly associated with all growth and adiposity outcomes at each time point. This single item representing *slowness in eating* was used in subsequent analyses and the distribution of responses is shown in Table 3.

**Figure 2.**
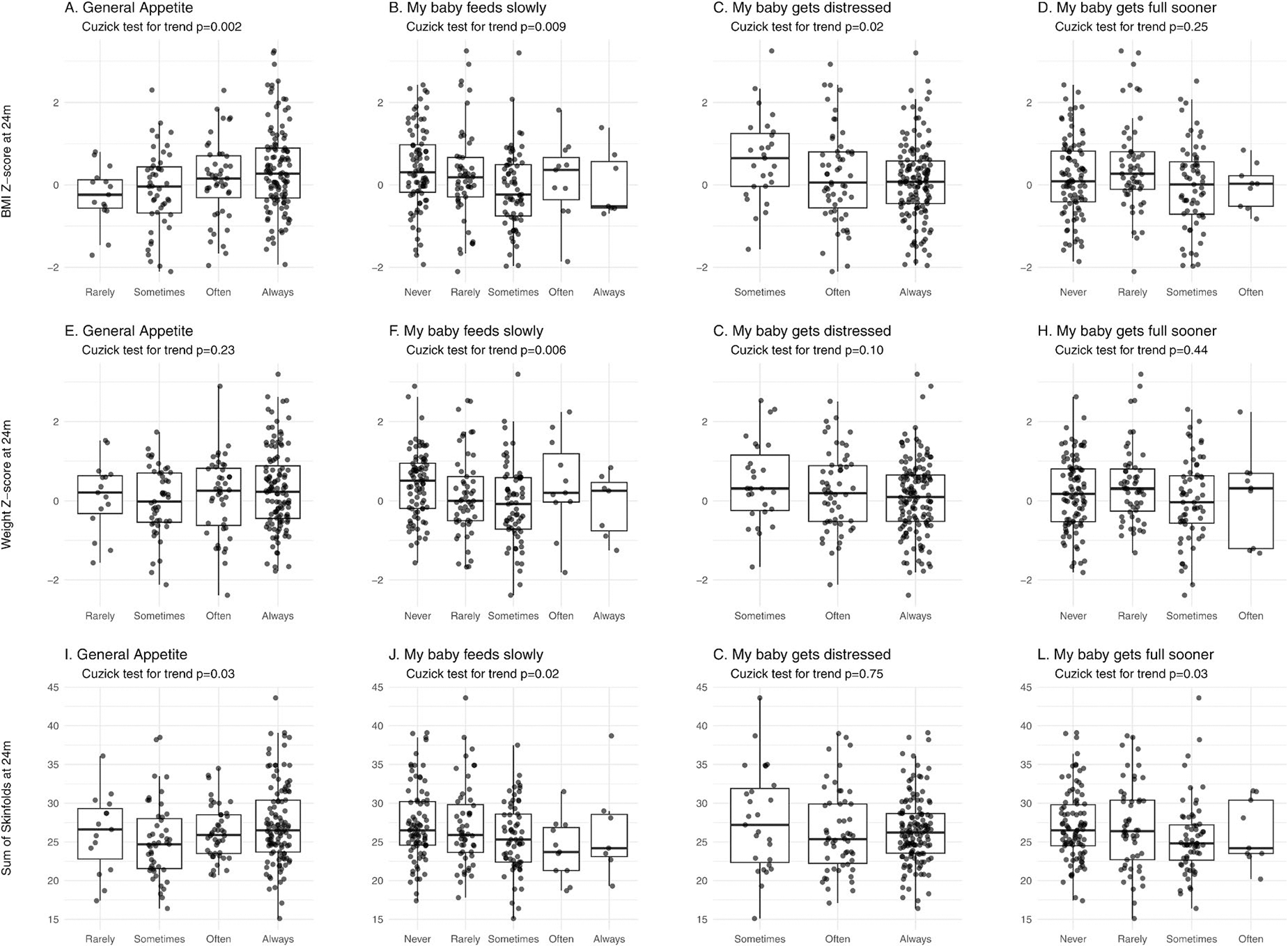
Relationship between selected BEBQ items assessed at age 3 months with growth and adiposity outcomes measured at 24 months of age. A. BMIZ vs. General Appetite; B. BMIZ vs *My baby feeds slowly*; C. BMIZ vs *My baby gets distressed while feeding*; D. BMIZ vs *My baby gets full before taking all the milk I think s/he should have*; E. WAZ vs. General Appetite; F. WAZ vs *My baby feeds slowly*; G. WAZ vs *My baby gets distressed while feeding*; H. BMIZ vs *My baby gets full before taking all the milk I think s/he should have*; I. Sum of skinfolds vs. General Appetite; J. Sum of skinfolds vs *My baby feeds slowly*; K. Sum of skinfolds vs *My baby gets distressed while feeding*; L. Sum of skinfolds vs *My baby gets full before taking all the milk I think s/he should have*. The Cuzick test for trend was used to test the significance of the overall trend across categories.

As our main outcome of interest was BMIZ at 24m, we first tested for trends across categories for *general appetite*, and the single items described above that are related to *slowness in eating, enjoyment of food* and *satiety responsiveness.* Cuzick’s test for trend revealed a significant positive trend across BEBQ *general appetite* scores with BMIZ at 24m (*p* = 0.002, Figure 2A) and negative trends across *eating slowly* (*p* = 0.009, Figure 2B) and *distressed while feeding* (*p* = 0.02, Figure 2C). Results for the secondary outcomes, WAZ and sum of skinfolds at 24m are shown in Figures 2D through I.

**Figure 3.**
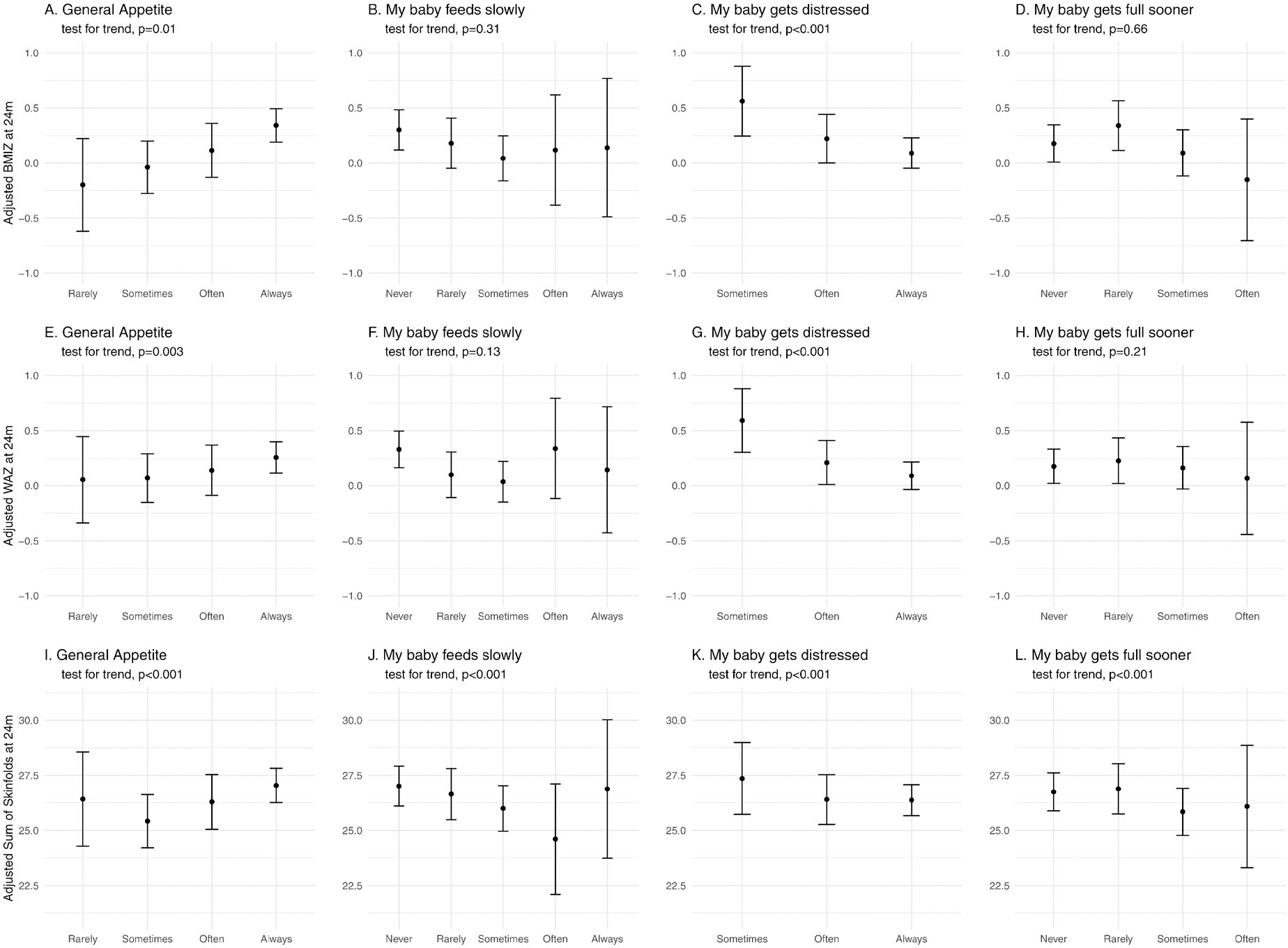
Relationship between selected BEBQ items assessed at age 3 months with growth and adiposity outcomes measured at 24 months of age, adjusted for growth/adiposity at 3 months. Shown are adjusted means and 95% confidence intervals from regression models, with a linear test for trend. A. BMIZ vs. General Appetite; B. BMIZ vs *My baby feeds slowly*; C. BMIZ vs *My baby gets distressed while feeding*; D. BMIZ vs *My baby gets full before taking all the milk I think s/he should have*; E. WAZ vs. General Appetite; F. WAZ vs *My baby feeds slowly*; G. WAZ vs *My baby gets distressed while feeding*; H. BMIZ vs *My baby gets full before taking all the milk I think s/he should have*; I. Sum of skinfolds vs. General Appetite; J. Sum of skinfolds vs *My baby feeds slowly*; K. Sum of skinfolds vs *My baby gets distressed while feeding*; L. Sum of skinfolds vs *My baby gets full before taking all the milk I think s/he should have.*

To consider the possibility that the associations just described may be due to the long term effects of growth and adiposity at age 3m on growth and adiposity outcomes at 24m (e.g. larger babies remain larger), we repeated the analyses using regression analysis to adjust for growth and adiposity at 3m and examined the trends in adjusted means. After adjusting for BMIZ at 3m, the linear trend in BMIZ at 24m across *general appetite* (*p* = 0.014) and *distressed while feeding (p* < 0.001*)* categories was significant, but the association of *eats slowly* with BMIZ at 24m was attenuated and not statistically significant (Figure 3A–C). Similar patterns were observed for WAZ at 24m adjusted for WAZ at 3m (Figure 3D–F). However, for the sum of skinfolds, all appetite traits and items showed significant trends in the expected directions when values at 24m were adjusted for values at 3m (Figure 3G–I).

### Associations between BEBQ measures and maternal and nutritional exposures

Maternal weight status based on early-pregnancy BMI, pregnancy weight gain, and birthweight Z-score were not associated with any BEBQ outcomes, even after adjustment for prenatal weight (data not shown).

**Figure 4.**
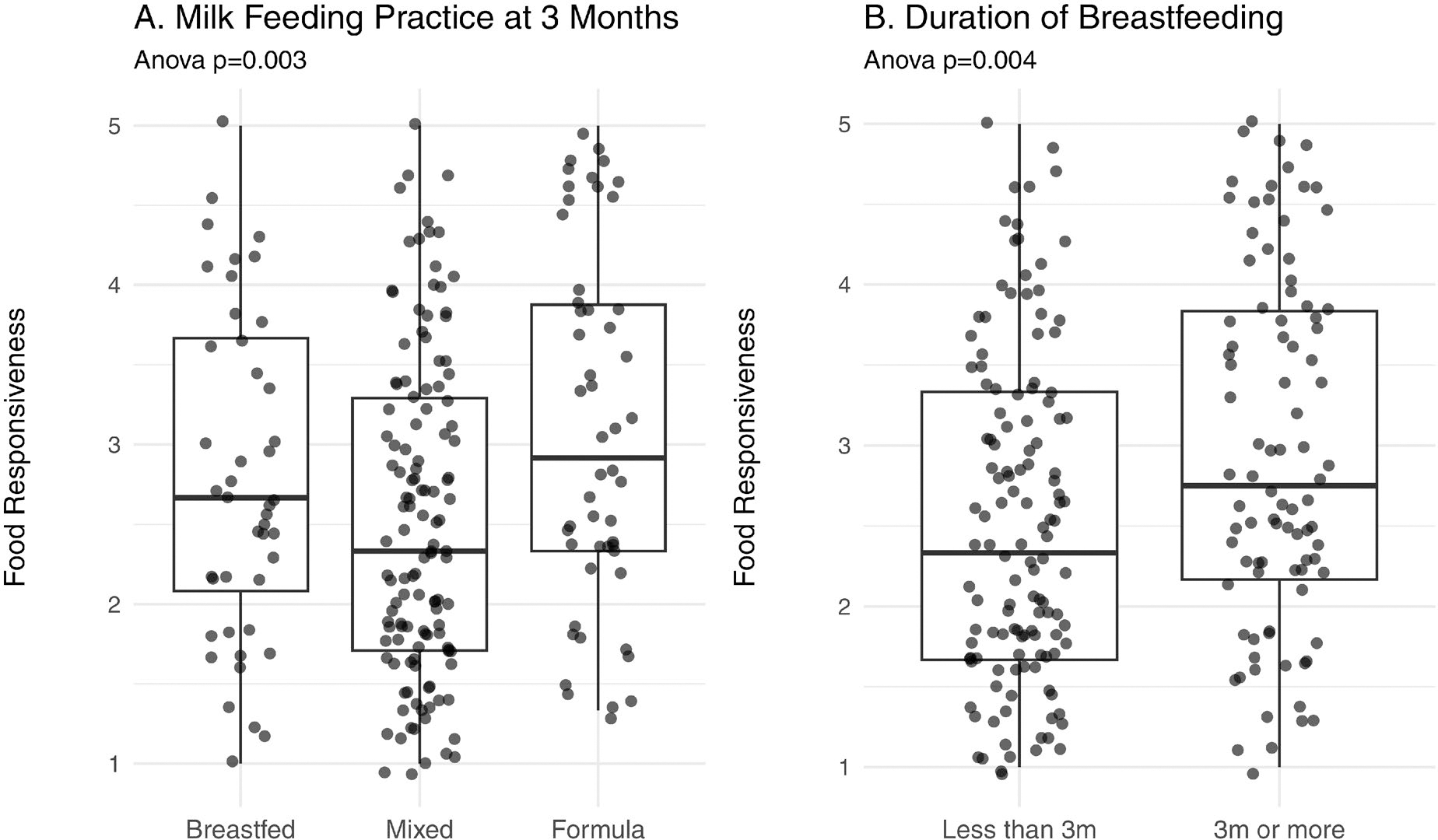
Relationship between the appetitive trait, *Food Responsiveness,* and feeding patterns: A. Milk feeding practice at 3 months; and B. Duration of breastfeeding.

Milk feeding practice *(*breast, formula, mixed) at 3m was significantly (*p* = 0.003) associated with *Food responsiveness* scores (Figure 4a). In pairwise comparisons with Bonferroni correction, the statistically significant difference was between the formula-fed (mean score = 2.5 ± 1.0) and mixed-fed (mean score = 3.1 ± 1.1) groups (adjusted *p* = 0.004). Milk feeding practice was not associated with the other appetitive traits. Duration of breastfeeding (months) was not associated with any appetitive traits, but mothers who stopped breastfeeding at or before 3m vs. those who continued breastfeeding beyond 3m reported lower *food responsiveness* scores (*p* = 0.006, Figure 4B.

Age at introduction to solids was not associated with any BEBQ measures. However, parents who reported frequently adding cereal to the bottle at 3m were more likely to rate their infants as faster eaters (*p* = 0.003). *Food responsiveness* was significantly higher on average for infants of mothers who experienced food insecurity (mean = 2.9) versus those who did not (mean = 2.60, *p* = 0.05). In addition, mothers who reported food insecurity were more likely to report that their infants were sometimes-always distressed while feeding (*p* = 0.002), but there were no differences in infant growth/adiposity outcomes based on reported food insecurity.

### Associations between growth and adiposity outcomes with BEBQ measures, maternal and nutritional exposures

In stepwise multiple regression, multiple indicators of infant appetitive traits were significantly associated with BMIZ at 24m after accounting for BMIZ at 3m (Table 4), namely “My baby eats slowly” (negative association), “My baby gets distressed when feeding” (negative association), and *General appetite* (positive association). These indicators were selected in the stepwise model after maternal obesity status, frequency of adding cereal to the bottle, birthweight Z-score and exclusive breastfeeding at 3m. For WAZ at 24m, “My baby gets distressed when feeding” was a significant predictor after accounting for WAZ at 3m, maternal obesity and exclusive breastfeeding at 3m. Appetitive traits were not associated with the sum of skinfolds after accounting for covariates.

## Discussion

This large, prospective, longitudinal study examined relationships between early life appetitive traits and measures of growth and adiposity through 24m of age among healthy, term African-American infants. In this sample, only the BEBQ *food responsiveness* appetitive trait construct demonstrated internal consistency. However, some individual questions from the BEBQ showed persistent, longitudinal associations with infant growth. Specifically, we found that maternal assessments of *general appetite, eating slowly* and *distress while feeding* reported at age 3m, when infants were on a nearly exclusive liquid milk diet, were correlated with BMIZ, WAZ and sum of skinfolds at 24m. In part, this was due to “tracking,” the fact that infants with greater relative weight and adiposity at 3m may continue to be larger at 24m. When adjusted for size at 3m, these relationships remained significant for *general appetite* and an indicator of *enjoyment of food*.

The association of baby’s *general appetite* with BMIZ was noted in the original study of the BEBQ (Llewellyn et al. 2011) conducted in a British cohort and in subsequent studies with varied racial and ethnic composition that were predominated by participants who identified as White (Barrett and Thompson 2022; van Jaarsveld et al. 2011; Kong et al. 2020). Importantly, our finding that the association of BMIZ and *general appetite* remained significant at 24m following adjustment for BMIZ at 3m indicating that for two children with the same BMIZ at 3m, the one with a higher appetite score had a greater BMIZ at 24m than the child with a lower score. Indeed, *general appetite*, along with responses to other single items reflecting appetitive behaviours (“My baby eats slowly” and “My baby gets distressed when eating’) were associated with BMIZ at 24m after accounting for other factors associated with childhood obesity, namely maternal obesity, adding cereal to the bottle, birthweight Z-score and exclusive breastfeeding at 3m. Additionally, *general appetite* ratings associated significantly and positively with skinfold thickness – a measure of subcutaneous adiposity – at 12 and 24m, although this association did not persist in multivariable models. The association of *general appetite* with skinfold measures is consistent with that of Patel et al. (2018) who reported that the BEBQ general appetite item was positively correlated with infant skinfold thickness at 6m of age. Of note, Patel et al. is the only prior study that specifically recruited women with obesity and tested associations between BEBQ-measured infant appetitive traits and infant skinfold thicknesses. Though an imperfect measure of fatness, skinfolds offer important information about degree of adiposity not captured by weight-for-age and BMI.

Consistent with this study’s hypotheses and prior reports (Kininmonth et al. 2021; Vandyousefi et al. 2021; Quah et al. 2015; Mallan et al. 2014; Shepard and Chandler-Laney 2015), there was a negative trend across slowness in eating (the item “my baby feeds slowly”) responses with infant BMIZ, WAZ, and skinfold thickness at 12 and 24m in unadjusted models; caregivers who reported that their babies never ate slowly had higher values for growth and adiposity. After adjusting for size at 3m, the negative trends for slowness in eating were attenuated for BMIZ and WAZ. However, in multivariable models for BMIZ at 24m, “my baby eats slowly” was a significant and independent predictor when combined with other appetitive traits and covariates associated with infant weight gain.

We also observed negative associations between growth and/or adiposity at 24m and single item questions related to “distress when feeding” (part of the *enjoyment of food* appetitive trait construct) and getting full easily (part of the *satiety responsiveness* appetitive trait construct). Interestingly, these negative trends became more pronounced after adjusting for growth/adiposity measures at 3m, and “distress when feeding” remained significant in the multivariable models for BMIZ and WAZ at 24m in combination with other covariates.

A novel feature of this study’s design was the approximately equal distribution of mothers who had a BMI in the obese or healthy weight range at the beginning of their pregnancy. To our knowledge, no other studies have examined whether early appetitive traits of infants differ according to maternal weight status. Maternal obesity is a risk factor for obesity in offspring (Heslehurst et al. 2019), however the various mechanisms and potential pathways underlying this association are complex and not well characterised. Physiologic mechanisms driving this association could be explained by shared genetics or long-lasting effects of the intrauterine milieu on infant appetitive behaviours (Thompson 2013). Alternatively, feeding practices may be shaped by maternal perceptions of infant appetitive traits (Thompson et al. 2014) and infant appetitive behaviours may have a genetic influence (Llewellyn et al. 2012). Infants of mothers in both groups were equally likely to eat slowly, have a big appetite, and be responsive to milk and feeding cues. However, in multivariable models, maternal obesity status and appetitive traits were both independent predictors of BMIZ and WAZ at 24m. Our finding suggests that maternal obesity status may be a marker for other genetic and/or environmental influences on weight gain in the first 2 years of life that are independent of early infant appetitive traits or maternal perceptions of their infant’s appetitive traits.

Key features of the postnatal nutritional environment include nutrition-related exposures such as type of milk provided (human milk vs formula), the duration of breastfeeding, timing of introduction of solids, and so on. Mothers and other caregivers play a key role in feeding in early life, and perceptions of an infant’s appetitive traits might shape decisions around when, how, and what to feed. For example, in a study by Mallan et al. (Mallan et al. 2014), BEBQ traits associated significantly with milk feeding practice (breast, formula, or mixed), with formula fed infants having the lowest *food responsiveness* scores. In our study, babies fed both formula and breastmilk had higher *food responsiveness* ratings than formula-fed babies. In addition, *food responsiveness* scores were significantly and positively correlated with duration of breastfeeding; specifically, mothers who continued to breastfeed their babies beyond 3m (when the BEBQ was administered) rated their babies higher on *food responsiveness* than mothers who stopped at or before 3m. This could indicate that mothers who perceived their babies as responsive to breastfeeding continued this mode of feeding relatively longer, or perhaps breastfeeding (whether exclusive or as part of a mixed-milk diet) is conducive to the development of food responsiveness in infants. Alternatively, mothers experiencing difficulties with breastfeeding may have rated their infants as less food responsive and been more likely to stop breastfeeding earlier. Interestingly, Vandyousefi et al. (Vandyousefi et al. 2022), recently reported that low-income Hispanic mothers in New York City were more likely to breastfeed longer if they perceived their infants to be slow eaters. Various sociocultural and economic factors undoubtedly shape mothers’ feeding choices as well as their perceptions of children’s appetite. Indeed, lower rates and shorter duration of breastfeeding are risk factors for obesity that disproportionately impact the African-American community (Roess et al. 2018; Thompson and Bentley 2013), and in this study, feeding mode was an independent predictor of WAZ at 24m in addition to the appetitive trait item of distress when feeding. Another potential risk factor for obesity is adding cereal to the baby’s bottle (Appleton et al. 2018), which in the current study was found to occur more frequently among mothers who rated their infants as “faster” eaters. Frequency of adding cereal to the bottle and “My baby eats slowly” were independent predictors of BMIZ at 24m in multivariable models.

Consideration of household food insecurity was another novel aspect of this study. Families in food insecure households are “at times unable to acquire adequate food for one or more household members because they had insufficient money and other resources for food” (Coleman-Jensen et al. 2021). Food insecurity is a potential risk factor for the development of obesity, but the evidence remains mixed (St Pierre et al. 2022) and we did not find an association between food insecurity and infant growth or adiposity. In this sample, 38% of mothers reported experiencing some degree of food insecurity in their 3^rd^ trimester. These mothers rated their infants higher on *food responsiveness* than mothers who did not report food insecurity in their household. There is evidence that food security status relates to caregiver feeding practices and beliefs around feeding (Orr et al. 2019; Gross et al. 2012). For example, Orr et al. (Orr et al. 2019) found that caregivers in food-insecure homes were more likely to immediately feed their infant in response to crying, and to believe this was the best way to stop an infant from crying. Further research is needed to understand how food insecurity shapes mother’s perception of infant’s appetite and feeding practices.

This study has numerous strengths. For the first time, BEBQ measures were analysed in a large cohort of African-American mothers and their infants followed for 24m. All participants completed the BEBQ at 3m, whereas other studies collected BEBQ data retrospectively (Vandyousefi et al. 2022; Sanjeevi et al. 2022; van Jaarsveld et al. 2011), when answers may be confounded by the fact that infants are no longer primarily milk-feeding. By design, this study included women with a pre-pregnancy BMI in obese vs. healthy weight ranges, so as to explore the impact of maternal weight status on infant feeding behaviours. The longitudinal nature of the data allowed for testing prospective associations between BEBQ scores and the main outcome of interest, BMIZ, at 24m, while accounting for other early life factors known to be associated with infant growth and adiposity, such as duration of breastfeeding and timing of introduction of solids. Limitations include the study’s observational nature, which precluded us from inferring causality. Due to the generally poor fit of the original BEBQ factor structure to the current dataset, we explored associations with other single items from the BEBQ survey. Other authors also found that the original BEBQ structure fit poorly to their data (Barrett and Thompson 2022; Oyama et al. 2021; Quah et al. 2015; Mallan et al. 2014), suggesting that the poor fit in this cohort is not unique to African-American mothers. Mother-infant dyads were recruited from a single urban hospital in the U.S., thus findings may not be generalisable to African-American mothers in other regions and from other socioeconomic backgrounds. Findings also may not be generalisable to mothers who are not African-American. Variation in sample characteristics like socioeconomic status, ethnicity, and cultural norms may have contributed to poor reproducibility across studies (Oyama et al. 2021). Finally, it was not possible to perform a direct test of convergent validity, for example by testing associations between BEBQ scores and direct observations of infant eating behaviours.

## Conclusions

This large, longitudinal study among African-American mother-infant dyads found that *general appetite* of infants and other single items from the BEBQ were significantly correlated with BMIZ, WAZ, and skinfold thickness at 12 and 24m, but not linear growth. The correlations were modest in magnitude yet similar to those reported in other studies and persistent to 24m, even after adjusting for other important factors such as tracking (i.e. BMIZ at 3m), maternal obesity status, milk feeding practice and adding cereal to the bottle. Further effort is needed to develop a culturally appropriate BEBQ instrument for African-Americans that accurately captures the different dimensions of appetitive traits in this life stage. Such an instrument would strengthen future investigations of factors that influence feeding decisions by caregivers and more accurately identify characteristics in early infancy that may lead to excess or inadequate weight.

## Supplementary Material

Supp 1

Supplemental data for this article can be accessed online at https://doi.org/10.1080/03014460.2025.2557266.

## Figures and Tables

**Table 1.** Characteristics of infant Growth and Microbiome Study mother-offspring dyads included in analyses of associations among appetitive traits, intrauterine and post-natal nutritional exposures, and growth/adiposity markers in the first 2 years of life (n = 222, unless otherwise noted).

Mothers	

Age (years), median (IQR)	25.6 (22.1, 29.0)
Education, n (%)	
Some high school	25 (11%)
Finished high school/GED	58 (26%)
Some business/trade/technical school	15 (7%)
Completed business/trade/technical school	41 (18%)
Some college	69 (31%)
Finished college/attended or completed graduate school	14 (6%)
Household gross income per year (dollars), n (%)	
Less than 10,000	36 (16%)
10,000–49,999	125 (56%)
50,000–89,999	41 (18%)
≥90,000	14 (6%)
Don’t know	6 (3%)
Food insecure in the 3^rd^ trimester, n (%)	82 (37%) (*n* = 221)
Early pregnancy weight (pounds), median (iQR)	179 (129, 234) (*n* = 221)
Maternal weight status, n (%)	
Group with healthy weight (<25 kg/m^2^)	102 (46%)
Group with obesity (>30 kg/m^2^)	120 (54%)
Maternal gestational weight gain (pounds), mean ± SD	24.6 ± 17.0 (*n* = 221)
**Infants**	
Age (months), median (IQR)	
3-month visit	3.0 (2.9, 3.1) (*n* = 222)
12-month visit	12.0 (11.9, 12.1) (*n* = 220)
24-month visit	24.0 (23.9, 24.2) (*n* = 222)
Female, n (%)	116 (52%)
Birth weight (kg), median (IQR)	3.2 (3.0, 3.5)
WHO Birth weight z-score, median (IQR)	−0.18 (−0.70, 0.55)
Gestational age (weeks), mean ± SD	39 ± 1.1
Exclusive breastfeeding*, n (%)	
Never	161 (73%)
1–6 months	36 (16%)
>6 months	25 (11%)
Duration of breastfeeding (months), median (IQR)	2.04 (0.46, 7.89) (*n* = 218)
Milk feeding practices at 3 months, n (%)	
Breastmilk	44 (20%)
Formula	126 (57%)
Mixed Milk Feeding	52 (23%)
Mothers breastfeeding (exclusively or mixed milk feeding) beyond 3 months, n (%)	90 (41%) (*n* = 218)
Age at introduction to solid foods (months), mean ± SD	3.6 ± 1.7
Cereal frequently added to bottle at 3 months, n (%)	66 (30%)
WHO Weight-for-age z-score, mean ± SD	
3-month visit	−0.14 ± 0.90 (*n* = 222)
12-month visit	0.23 ± 1.06 (*n* = 220)
24-month visit	0.18 ± 0.97 (*n* = 222)
WHO Body mass index-for-age z-score, median (IQR)	
3-month visit	0.07 (−0.57, 0.75) (*n* = 222)
12-month visit	0.31 (−0.57, 1.08) (*n* = 220)
24-month visit	0.11 (−0.44, 0.75) (*n* = 222)
WHO Height-for-age z-score, mean ± SD	
3-month visit	−0.34 ± 0.97 (*n* = 222)
12-month visit	0.00 ± 1.02 (*n* = 220)
24-month visit	0.08 ± 1.01 (*n* = 222)
Sum of skinfolds (mm), median (IQR)	
3-month visit	31.2 (27.70, 34.90) (*n* = 221)
12-month visit	27.0 (24.40, 31.05) (*n* = 220)
24-month visit	25.9 (23.20, 29.70) (*n* = 219)

* At each visit, mothers were asked if they were giving their infants breastmilk, formula or both, and exclusive breastfeeding was based on this question only. Other items, such as water or complementary foods were not considered in this categorisation.

**Table 2.** Associations between Baby Eating Behaviour Questionnaire appetitive trait constructs and their individual items with growth and adiposity outcomes at 3, 12 and 24 months of age.

	BMI Z-score^1^			Weight-for-age Z-score^1^			Height-for-age Z-score^1^			Sum of Skinfolds		
Baby Eating Behaviour Questionnaire Constructs and Items	3M	12M	24M	3M	12M	24M	3M	12M	24M	3M	12M	24M

**Food responsiveness**^2^ **(Cronbach's α = 0.84)**	0.02	0.12	0.06	0.02	0.11	0.06	0.00	0.05	0.03	−0.03	0.09	0.01
My baby frequently wants more milk than 1 provide	−0.06	0.06	0.02	−0.06	0.09	0.09	−0.02	0.12	0.12	−0.04	0.09	−0.08
If allowed to, my baby would take too much milk	0.04	0.11	0.13	0.05	0.12	0.12	0.04	0.05	0.05	−0.01	0.08	0.04
Even when my baby has just eaten well he/she is happy to feed again if offered	0.04	0.10	0.02	0.05	0.07	0.01	−0.01	0.00	−0.02	−0.01	0.08	0.04
My baby is always demanding	0.00	0.07	−0.03	−0.03	0.07	0.00	−0.03	0.03	0.05	0.01	0.04	−0.05
If given a chance, my baby would always be feeding	0.01	0.08	0.05	0.01	0.07	0.00	0.02	0.00	−0.04	−0.06	0.05	−0.02
My baby can easily take a feed within 30min of the last one	0.05	**0.15** *	0.12	0.03	0.12	0.08	−0.02	0.01	−0.01	0.00	0.09	0.10
**General appetite** ^3^	0.08	**0.15** *	**0.20** *	0.04	0.10	0.08	−0.02	−0.04	−0.06	0.07	**0.21** *	**0.15** *
**Enjoyment of Food (Cronbach's α = 0.39)**	−0.03	−0.15*	−0.14*	0.00	−0.08	−0.14*	0.06	0.04	−0.07	0.10	−0.08	−0.02
My baby seems contented while feeding	0.00	−0.01	0.01	−0.02	−0.01	−0.03	−0.02	−0.02	−0.06	0.02	0.00	0.02
My baby loves milk	−0.09	0.05	−0.03	−0.06	0.02	−0.08	0.03	−0.05	−0.07	0.02	0.08	0.07
My baby becomes distressed while feeding (inverse scoring)	−0.01	−0.15*	−0.14*	0.03	−0.06	−0.11	0.08	0.08	−0.01	0.07	−0.07	0.00
My baby enjoys feeding time	−0.06	−0.07	−0.13	−0.01	−0.04	−0.17*	0.02	0.00	−0.12	0.12	−0.05	−0.01
**Slowness in eating (Cronbach's α=0.51)**	−0.11	−0.16*	−0.23*	−0.09	−**0.13**	−0.16*	−0.04	−0.04	−0.03	−0.16*	−0.15*	−0.19*
My baby finishes feeding quickly (inverse scoring)	−0.04	−0.10	−0.16*	−0.08	−0.11	−0.15*	−0.10	−0.11	−0.09	−0.09	−0.09	−0.06
My baby takes more than 30min to finish feeding	−0.10	−0.10	−0.14*	−0.09	−0.10	−0.12	−0.04	−0.04	−0.05	−0.16*	−0.13	−0.15*
My baby feeds slowly	−0.15*	−0.16*	−0.19*	−0.15*	−0.20*	−0.20*	−0.07	−0.14*	−0.10	−0.16*	−0.19*	−0.16*
My baby sucks more and more slowly during the course of the feed	0.02	−0.03	−0.09	0.05	0.04	0.00	0.03	0.06	0.06	−0.04	−0.01	−0.12
**Satiety responsiveness (Cronbach's α = 0.46)**	−0.08	−0.03	−0.06	−0.09	−0.08	−0.03	−0.06	−0.04	0.05	−0.14*	−0.11	−0.16*
My baby gets full up easily	−0.12	0.01	−0.02	−0.08	−0.01	0.01	0.01	0.03	0.07	−0.07	−0.05	−0.05
My baby gets full before taking all the milk 1 think he/she should have	−0.04	−0.04	−0.07	−0.05	−0.08	−0.05	−0.04	−0.07	0.02	−0.14*	−0.14*	−0.14*
My baby finds it difficult to manage a complete feed	0.03	−0.01	−0.03	−0.04	−0.05	−0.02	−0.13*	−0.04	0.01	−0.07	0.00	−0.17*

* P<=0.05.

1 Z-score calculated using the WHO reference.

2 Confirmatory factor analysis showed internal consistency.

3 General appetite is a single item appetitive trait. There were only 4 responses of "never," so "never" and "rarely" were combined for use in analyses.

**Table 3.** Distribution of Baby Eating Behaviour Questionnaire appetitive trait constructs and individual items.

Distribution of responses for BEBQ items	

General appetite	
Never	4 (2%)
Rarely	11 (5%)
Sometimes	47 (21%)
Often	44 (20%)
Always	116 (52%)
Slowness in eating^1^	
Never	84 (38%)
Rarely	53 (24%)
Sometimes	67 (30%)
often	11 (5%)
Always	7 (3%)
Food responsiveness^2^	
Never	65 (29%)
Rarely	73 (33%)
Sometimes	48 (22%)
often	33 (15%)
Always	3 (1%)
Enjoyment of food^2^	
Sometimes	6 (3%)
often	103 (46%)
Always	113 (51%)

1 Based on the individual item, “My baby eats slowly.”

2 Appetitive trait construct score shown as integer values rather than continuous (e.g. scores for “Never” are 1.0 to 1.9).

**Table 4.** Stepwise regression analysis predicting BMIZ, weight z and sum of skinfolds at 24 months with appetitive traits and other predictors of infant weight gain.

	*Variable*	*Coefficient* [95% CI]	P>|t|	*Adjusted R^2^*

*BMI Z at 24m (n = 217)*	BMI Z at 3m	0.51 [0.40, 0.61]	<0.001	0.42
	Maternal obesity	0.33 [0.12, 0.54]	0.002	
	Cereal in bottle	−0.28 [−0.52, −0.03]	0.026	
	Birthweight Z	0.13 [0.01, 0.25]	0.039	
	Exclusive breastmilk	−0.38 [−0.65, −0.11]	0.006	
	Distressed when feeding	−0.21 [−0.36, −0.06]	0.006	
	My baby eats slowly	−0.12 [−0.22, −0.02]	0.016	
	General appetite	0.19 [0.08, 0.29]	0.001	
	Constant	0.22 [−0.76, 1.21]	0.658	
*Weight Z at 24m (n = 217)*	Weight Z at 3m	0.70 [0.59, 0.81]	<0.001	0.45
	Maternal obesity	0.31 [0.12, 0.51]	0.002	
	Exclusive breastmilk	−0.41 [−0.66, −0.17]	0.001	
	Distressed when feeding	−0.22 [−0.36, −0.09]	0.002	
	Constant	0.90 [0.18, 1.61]	0.015	
*Σ skinfolds at 24m (n = 214)*	Σ skinfolds at 3m	0.44 [0.34, 0.54]	<0.001	0.27
	Birthweight Z	1.06 [0.40, 1.71]	0.002	
	Pregnancy weight gain	−0.05 [−0.08, −0.01]	0.007	
	Constant	13.89 [10.57, 17.21]	<0.001	

Stepwise model for each dependent variable included the value at 3 months (required), and selection with a required p-value for retention in the model of *p* ≤ 0.05 among the following variables: Maternal obesity (yes vs no), pregnancy weight gain, birthweight Z-scores, feeding mode at 3 months (breastmilk only, formula only, or both breastmilk and formula), duration of breastfeeding for 3 or more months (yes vs no), age at introduction of solids, how frequently cereal was added to the bottle at 3 months (never vs. often), food insecurity, *General Appetite, Food Responsiveness, My Baby Eats Slowly, My baby becomes distressed when feeding, My baby gets full up easily.*

## Data Availability

Data is available from the corresponding author upon request, provided bioethical approval has been secured.
