# Supplementary material for: Early infant appetitive traits are associated with growth status and adiposity in African-American infants and toddlers
Source: Ann Hum Biol. Author manuscript; Available in PMC 2025 Nov 18. (PMC12626232; doi:10.1080/03014460.2025.2557266)
Supplement: Supp 1 [file NIHMS2116485-supplement-Supp_1.pdf]

Supplemental Table 1. Comparison of baseline characteristics of Infant Growth and Microbiome Study mother-offspring dyads who enrolled at birth, completed the Baby Eating Behavior Questionnaire[18] (BEBQ) at the 3-month visit, and had data for body mass index (BMI) z-score (BMIZ) at 24 months (n = 222) versus those who enrolled at birth but did not complete the BEBQ at the 3-month visit and/or did not have BMIZ data at 24 months (n = 146)

Factor	Enrolled infants not included in analyses (n=146)	Analytic Sample	p-value
N	146	222	
Mothers			
Age (years), median (IQR)	25.1 (22.1, 28.0)	25.4 (21.9, 28.8)	0.57
Education, n (%)			0.45
Some high school	18 (12.3%)	25 (11.3%)	
Finished high school/GED	52 (35.6%)	58 (26.1%)	
Some Business/Trade/Technical School	8 (5.5%)	15 (6.8%)	
Completed Business/Trade/Technical school	20 (13.7%)	41 (18.5%)	
Some college	37 (25.3%)	69 (31.1%)	
Finished college	9 (6.2%)	12 (5.4%)	
Some graduate school	1 (0.7%)	2 (0.9%)	
Finished graduate school	1 (0.7%)	0 (0.0%)	
Household gross income per year (dollars), n (%)			0.13
Less than 10,000	30 (20.5%)	36 (16.2%)	
10,000 – 49,999	76 (52.1%)	125 (56.3%)	
50,000 – 89,999	25 (17.1%)	41 (18.5%)	
≥90,000	4 (2.7%)	14 (6.3%)	
Don't know	10 (6.8%)	6 (2.7%)	
Missing	1 (0.7%)	0 (0.0%)	
Food insecure in the 3rd trimester, n (%)			0.67
No	95 (65.1%)	139 (62.6%)	
Yes	51 (34.9%)	82 (36.9%)	
Missing	0 (0.0%)	1 (0.5%)	
Early pregnancy weight (pounds), median (IQR)	163 (125, 226) (n=145)	181 (129, 234)	0.67
Maternal weight status, n (%)			0.45
Group with healthy weight (<25 kg/m2)	73 (50.0%)	102 (45.9%)	
Group with obesity (>30 kg/m2)	73 (50.0%)	120 (54.1%)	
Gestational weight gain (pounds), mean ± SD	23.8 ± 15.2 (n=144)	24.6 ± 17.0	0.65
Female, n (%)	69 (47.3%)	116 (52.3%)	0.35

Information Classification: General

Birth weight (kg),median (IQR)	3.26 (2.92, 3.58)	3.23 (2.95, 3.55)	0.65
WHO Birth weight z-score, median (IQR)	-0.29 (-1.04, 0.28)	-0.32 (-0.91, 0.25)	0.77
Gestational age (weeks), mean ± SD	39.40 ± 1.11	39.36 ± 1.14	0.77

Information Classification: General

Supplemental Table 2. Comparison of characteristics for those with complete data vs. those with 1 or more missing data points.

	Complete Data	≥1 missing data points	p-value
N	212	10	
Mother			
Age (years), median (IQR)	25.5 (21.9, 28.6) (n=212)	24.5 (20.3, 34.3) (n=10)	0.95
Education, n(%)			0.06
Some high school	23 (10.8%)	2 (20.0%)	
Finished high school/GED	57 (26.9%)	1 (10.0%)	
Some Business/Trade/Technical School	14 (6.6%)	1 (10.0%)	
Completed Business/Trade/Technical school	39 (18.4%)	2 (20.0%)	
Some college	66 (31.1%)	3 (30.0%)	
Finished college	12 (5.7%)	0 (0.0%)	
Some graduate school	1 (0.5%)	1 (10.0%)	
Household gross income per year (dollars), n(%)			0.58
Less than 10,000	34 (16.0%)	2 (20.0%)	
10,000 – 49,999	121 (57.1%)	4 (40.0%)	
50,000 – 89,999	39 (18.4%)	2 (20.0%)	
≥90,000	13 (6.1%)	1 (10.0%)	
Don't know	5 (2.4%)	1 (10.0%)	
Food insecure in the 3rd trimester, n (%)			0.81
Not food insecure	133 (62.7%)	6 (60.0%)	
Food insecure	79 (37.3%)	3 (30.0%)	
Missing	0 (0.0%)	1 (10.0%)	
Early pregnancy weight (pounds), median (IQR)	181 (129.5, 234) (n=212)	183 (129, 231) (n=10)	0.97
Maternal weight status, n (%)			0.79
Lean	97 (45.8%)	5 (50.0%)	
Obese	115 (54.2%)	5 (50.0%)	
Maternal gestational weight gain (pounds), mean ± SD	24.4 ± 17.0 (n=212)	30.3 ± 15.7 (n=10)	0.28
Infants			
Age (months), median (IQR)			
3-month visit	3.0 (2.9, 3.1) (n=212)	3.0 (3.0, 3.1) (n=10)	0.55
12-month visit	12.0 (11.9, 12.1) (n=212)	11.9 (11.9, 12.2) (n=8)	0.46

Information Classification: General

24-month visit	24.0 (23.9, 24.2) (n=212)	24.1 (24.0, 24.2) (n=10)	0.17
Infant sex			0.15
Female	113 (53.3%)	3 (30.0%)	
Male	99 (46.7%)	7 (70.0%)	
Birth weight (kg), median (IQR)	3.23 (2.97, 3.55) (n=212)	3.03 (2.76, 3.24) (n=10)	0.09
WHO Birth weight z-score, median (IQR)	-0.29 (-0.91, 0.32) (n=212)	-0.58 (-1.46, -0.27) (n=10)	0.10
Gestational age (weeks), mean ± SD	39.4 ± 1.1 (n=212)	39.6 ± 1.2 (n=10)	0.50
Exclusive breastfeeding, n (%)			0.39
never BF	152 (71.7%)	9 (90.0%)	
1 to 6m	35 (16.5%)	1 (10.0%)	
>6m	25 (11.8%)	0 (0.0%)	
Duration of breastfeeding (months), median (IQR)	1.97 (0.5, 7.2) (n=213)	8.12 (2.1, 21.3) (n=5)	0.09
Milk feeding practices at 3 months, n (%)			0.64
Breastmilk	43 (20.3%)	1 (10.0%)	
Formula	119 (56.1%)	7 (70.0%)	
Mixed milk feeding	50 (23.6%)	2 (20.0%)	
Mothers breastfeeding (exclusively or mixed milkfeeding) beyond 3 months, n (%)			0.66
No	125 (59.0%)	3 (30.0%)	
Yes	87 (41.0%)	3 (30.0%)	
Missing	0 (0.0%)	4 (40.0%)	
Age at solid food introduction (months), mean ± SD	3.7 ± 1.7 (n=212)	3.1 ± 1.7 (n=10)	0.31
Cereal frequently added to bottle at 3 months, n (%)			0.49
Almost never	148 (69.8%)	8 (80.0%)	
Cereal frequently added to bottle	64 (30.2%)	2 (20.0%)	
WHO Weight-for-age z-score, mean ± SD			
3-month visit	-0.14 ± 0.90 (n=212)	-0.16 ± 1.02 (n=10)	0.95
12-month visit	0.22 ± 1.04 (n=212)	0.64 ± 1.46 (n=8)	0.27
24-month visit	0.16 ± 0.96 (n=212)	0.56 ± 1.27 (n=10)	0.21
WHO Body mass index-for-age z-score, median (IQR)			
3-month visit	0.08 (-0.56, 0.78) (n=212)	0.04 (-0.73, 0.24) (n=10)	0.78
12-month visit	0.29 (-0.57, 1.04) (n=212)	1.06 (-0.03, 2.12) (n=8)	0.17
24-month visit	0.09 (-0.45, 0.73) (n=212)	0.32 (-0.29, 1.59) (n=10)	0.23
WHO Height-for-age z-score, mean ± SD			

Information Classification: General

3-month visit	-0.34 ± 0.99 (n=212)	-0.43 ± 0.63 (n=10)	0.79
12-month visit	0.01 ± 1.02 (n=212)	-0.21 ± 0.97 (n=8)	0.55
24-month visit	0.08 ± 1.01 (n=212)	0.06 ± 1.06 (n=10)	0.96
Sum of skinfolds (mm), median (IQR)			
3-month visit	30.95 (27.60, 34.90) (n=212)	32.60 (31.40, 33.30) (n=9)	0.16
12-month visit	26.90 (24.20, 30.55) (n=212)	32.35 (27.20, 36.45) (n=8)	0.03
24-month visit	26.05 (23.30, 29.80) (n=212)	25.30 (20.60, 26.00) (n=7)	0.44

Data is missing for the following variables: Food insecurity (n=1), Duration of breastfeeding (n=4), 12 month visit (n=2), Sum of skinfolds at 3m (n=1) and 24m (n=3)

Information Classification: General

Supplemental Table 3. Comparison of BEBQ values for those with complete data vs. those with 1 or more missing data points.

	Complete Data<br>N = 212<br>Median (IQR)	≥1 missing data points<br>N = 10<br>Median (IQR)	p-value
**Food responsiveness**	2.5 (1.8, 3.5)	2.8 (2.7, 3.0)	0.26
My baby frequently wants more milk than I provide	2.0 (1.0, 3.0)	3.0 (2.0, 3.0)	0.16
If allowed to, my baby would take too much milk	3.0 (1.0, 4.0)	3.0 (3.0, 3.0)	0.74
Even when my baby has just eaten well he/she is happy to feed again if offered	3.0 (1.0, 3.0)	3.0 (2.0, 3.0)	0.52
My baby is always demanding	3.0 (2.0, 5.0)	3.0 (3.0, 5.0)	0.46
If given a chance, my baby would always be feeding	2.0 (1.0, 4.0)	3.0 (3.0, 3.0)	0.24
My baby can easily take a feed within 30 minutes of the last one	3.0 (2.0, 4.0)	3.0 (2.0, 4.0)	0.87
**General appetite**	5.0 (3.0, 5.0)	4.5 (3.0, 5.0)	0.97
**Enjoyment of Food**	5 (4.5, 5)	4.5 (4.5, 5)	0.12
My baby seems contented while feeding	5.0 (5.0, 5.0)	4.0 (4.0, 5.0)	0.01
My baby loves milk	5.0 (5.0, 5.0)	5.0 (5.0, 5.0)	0.21
My baby becomes distressed while feeding (inverse scoring)	5.0 (4.0, 5.0)	4.5 (4.0, 5.0)	0.49
My baby enjoys feeding time	5.0 (5.0, 5.0)	5.0 (5.0, 5.0)	0.85
**Slowness in eating**	2.2 (1.8, 2.8)	2.5 (2.2, 2.8)	0.33
My baby finishes feeding quickly (inverse scoring)	3.0 (2.0, 3.0)	3.0 (3.0, 3.0)	0.55
My baby takes more than 30 min to finish feeding	1.0 (1.0, 2.0)	2.0 (2.0, 2.0)	0.13
My baby feeds slowly	2.0 (1.0, 3.0)	2.0 (2.0, 3.0)	0.84
My baby sucks more and more slowly during the course of the feed	3.0 (1.0, 3.0)	3.0 (2.0, 3.0)	0.39
**Satiety responsiveness**	2.0 (1.7, 2.3)	2.2 (1.7, 2.3)	0.60

Information Classification: General

My baby gets full up easily	3.0 (2.0, 3.0)	2.0 (2.0, 3.0)	0.27
My baby gets full before taking all the milk I think he/she should have	2.0 (1.0, 3.0)	2.0 (1.0, 3.0)	0.64
My baby finds it difficult to manage a complete feed	1.0 (1.0, 2.0)	1.5 (1.0, 3.0)	0.08

Information Classification: General
